# Analysis of Effect of *Schisandra* in the Treatment of Myocardial Infarction Based on Three-Mode Gene Ontology Network

**DOI:** 10.3389/fphar.2019.00232

**Published:** 2019-03-20

**Authors:** Siyao Hu, Huali Zuo, Jin Qi, Yuanjia Hu, Boyang Yu

**Affiliations:** ^1^Jiangsu Key Laboratory of Traditional Medicine and Translational Research, China Pharmaceutical University, Nanjing, China; ^2^State Key Laboratory of Quality Research in Chinese Medicine, Institute of Chinese Medical Sciences, University of Macau, Taipa, Macau

**Keywords:** *Schisandra*, myocardial infarction, effect, three-mode network, gene ontology

## Abstract

*Schisandra chinensis* is a commonly used traditional Chinese medicine, which has been widely used in the treatment of acute myocardial infarction in China. However, it has been difficult to systematically clarify the major pharmacological effect of *Schisandra*, due to its multi-component complex mechanism. In order to solve this problem, a comprehensive network analysis method was established based-on “component–gene ontology–effect” interactions. Through the network analysis, reduction of cardiac preload and myocardial contractility was shown to be the major effect of *Schisandra* components, which was further experimentally validated. In addition, the expression of *NCOR2* and *NFAT* in myocyte were experimentally confirmed to be associated with *Schisandra* in the treatment of AMI, which may be responsible for the preservation effect of myocardial contractility. In conclusion, the three-mode gene ontology network can be an effective network analysis workflow to evaluate the pharmacological effects of a multi-drug complex system.

## Introduction

Cardiovascular disease, including atherosclerosis, myocardial infarction, heart failure and stroke, is the leading cause of morbidity and mortality in developed nations. Acute myocardial infarction (AMI) is induced through the narrowing of arteries caused by atherosclerotic plaques or the acute occlusion of the coronary artery by thrombosis, and has received extensive attention due to its high risk and poor outcome among all the symptoms of coronary heart disease (Holmes et al., 2011; Husted and Ohman, 2015).

Traditional Chinese medicine (TCM) is a commonly used therapeutic strategy for the treatment of AMI in China. And *Schisandra chinensis* is a commonly used TCM that has been clinically proven to alleviate the damage of myocytes after the onset of AMI. Many pharmacological research results have clarified the mechanisms of *Schisandra* (Li et al., 1996; Panossian and Wikman, 2008; Chang et al., 2013; Chen et al., 2013; Zhan et al., 2014). However, few studies have evaluated the major effects and possible mechanisms responsible for the treatment of AMI, due to the complexity of the multi-mechanisms associated with TCM (Gao et al., 2016; Tang et al., 2016). Thus, in order to research the multi-mechanism complex system in TCM, network pharmacology has been commonly used in recent years to predict the major target proteins or signal pathways of TCM.

In this study, network pharmacology was applied to analyze the major effect of *Schisandra*. In contrast to protein interaction networks, enriched gene ontology (GO) terms of AMI related genes were used to construct a gene ontology interaction (GOI) network, which can be used to simulate the functional interactions between differential expressed genes of disease. In general, this study aims to identify and validate major mechanism and related pharmacological effects of *Schisandra* in the treatment of AMI through a GOI network, which may offer a new method of network analysis to evaluate complex bio-systems.

## Materials and Methods

### Component Identification

For the construction of a “component–gene ontology” network of *Schisandra* in the treatment of AMI, the HPLC-Q-TOF-MS was used to analyze the *Schisandra* extract. Targets of *Schisandra* ingredients were further screened to enrich and construct the “component–gene ontology” network, which was a component for the integration of a three-mode network.

#### Sample Solution Preparation

To ensure the consistency of network analysis and experimental validation results, *Schisandra chinensis* Fructus of the same batch was utilized for the extraction, analysis and pharmacological experiment. *Schisandra chinensis* Fructus from *Schisandra chinensis* (Turcz.) Baill was obtained from Tianjin Tasly Pride Pharmaceutical Co. (Tianjin, China). The crude drug was extracted through a reflux condenser with 10 times the amount of distilled water at 100°C for 1 h. This procedure was repeated three times. The combined *Schisandra* extract was then concentrated under reduced pressure and dissolved through distilled water into an appropriate concentration for administration to mice. Deionized water was prepared using a Milli-Q Ultrapure water system (Millipore, Bedford, MA, United States). For analysis of the *Schisandra* component, dry extract was dissolved in 50% methanol and then centrifuged at 12000 rpm for 15 min. The supernatant was transferred to a 1.5 mL brown HPLC vial (Grace, Chicago, IL, United States) and stored at 4°C for analysis.

#### HPLC-Q-TOF-MS-MS Analysis Conditions

Chromatographic experiments were conducted on a Shimadzu Shimadzu (Kyoto, Japan) LC-2010 series. Chromatographic separation was performed on a Kromasil 100-5C18 (250 × 4.6 mm, 5 μm particle size) column, with the column temperature maintained at 30°C. The mobile phase was composed of solvent A (acetonitrile containing 0.01% v/v formic acid) and solvent B (ultrapure water containing 0.02% v/v acetic acid). The gradient elution conditions were: 0-15 min, 20% A; 15-25 min, 20-22% A; 25-45 min, 22-32% A; 45-65 min, 32-34% 75 min, 34-42% A; 75-95 min, 42-60% A; 95-110 min, 60-70% A; 110-125 min, 70-100% A; 125-130 min, 100% A. The injection volume was 15 μL. The elution rate was 0.8 mL/min and the detector was set at 203 nm.

The 6520 Q-TOF mass spectrometer (Agilent Technologies, Santa Clara, CA, United States) was equipped with an electrospray ionization (ESI) source. Ultrahigh purity argon was used as the collision gas and high purity nitrogen as the nebulizing gas. The following MS conditions were used: detector voltage was 1.65 kV, capillary voltage was 3.5 kV, heat block temperature was 325°C, nebulizer was 35 psig, nebulizing gas (N2) flow was 8.0 L/min, drying gas pressure (N2) was 72 kPa, ion trap pressure was 1.9 × 10^2^ Pa, TOF pressure was 2.2 × 10^4^ Pa, ion accumulation time was 100 ms. Scan ranges were set at m/z 100–1000 in both the positive and negative modes. The accurate mass determination was corrected by calibration using the sodium trifluoroacetate clusters as a reference. The peak area of molecular ion was then measured and normalized for the rough quantification of identified *Schisandra* components.

### Construction and Analysis of “Component-Gene Ontology-Effect” Three-Mode Network

**FIGURE 1:**
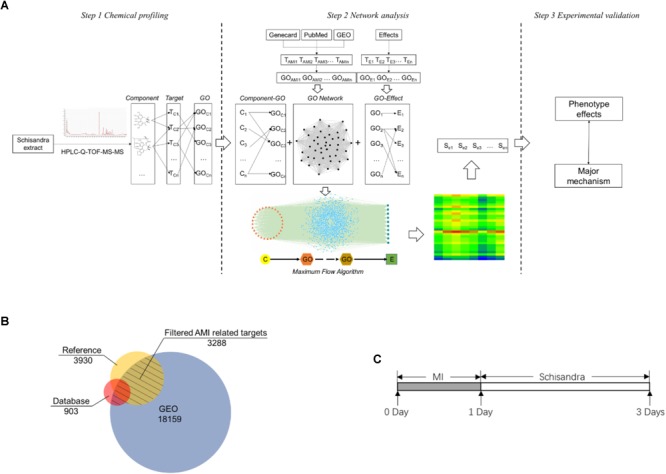
The *Schisandra* three-mode GOI network analysis workflow. **(A)** The components in the *Schisandra* extract were identified through HPLC-Q-TOF-MS-MS. Target proteins relevant to component (T_C_) were then obtained through PharmMapper. AMI related differential expressed genes (T_AMI_) and pharmacological effect related proteins (T_E_) were screened through data mining from three different databases **(B)**. After target data were enriched through the GO annotation database, the relationship between the component and pharmacological effects were connected through GO terms, and the “component–gene ontology–effect” three-mode network was constructed. The relation score between component and effect was calculated using a maximum flow algorithm. In the end, the prediction results were validated through pharmacological experiments **(C)**, with the corresponding drug given to mice one day after ligation of the left anterior descending.

In order to simulate and analyze the correlation between components and pharmacological effects, a three-mode network model, which included the pathological relationship between component and pharmacological effect, was constructed (Figure 1A).

#### Data Mining of Targets Related to *Schisandra* and AMI

##### Screening of identified Schisandra component’s related target

The chemical structures of *Schisandra* components identified through HPLC-Q-TOF-MS-MS were drawn using the ChemDraw 2004. After the structures were transformed into ^∗^.mol format, they were uploaded to the PharmMapper^1^ (Iyer et al., 2015) server to screen for component related drug-like target proteins. The filter species was set as *Mus Musculus*, and the top 300 drug-related proteins of each component were collected.

##### Myocardial infarction relevant targets

In order to reduce the bias of datamining, several different data sources were utilized to collect the AMI-related differentially expressed genes. The target data obtained from the public database would be more widely acknowledged. The differential expression genes collected from the GEO database ensures the precision of experimental animal species and organs. Target data screened from references of the PubMed database guarantees the inclusion of the latest research targets related to AMI.

For the screening of public disease databases, three data sources: GeneCard^2^, PharmGKB^3^, and the Therapeutic Target Database^4^ were searched using the keywords “Coronary heart disease OR Coronary artery disease OR Myocardial infarction OR Myocardial ischemia.” Snapshots of these databases were taken in December 2016. The collected target dataset was marked as *T_DB_*.

The PubMed database^5^ was then utilized for reference mining of the latest researched on AMI associated gene datasets, by screening the search terms “Coronary heart disease OR Coronary artery disease OR Myocardial infarction OR Myocardial ischemia.” Abstracts of reference within the past 5 years were downloaded and literature mining was carried out in order to collect the target data. After removing duplicates, the target dataset related to each pharmacological effect could be collected and was marked as *T_Ref_*.

The next step was datamining for data on differentially expressed genes (DEG) in the Gene Expression Omnibus (GEO^6^) database. The experimental terms of interest were evaluated by screening experimental designs. Designs that contained a model constructed by ligation of left anterior descending (LAD) surgery on the species of *Mus Musculus* were included, while raw data terms without enough duplications (>3) were excluded. The collected raw data (GSE775, GSE19322, and GSE49937) were then normalized by the RMA method through the Affy package in Bioconductor^7^. The significant DEGs between control and AMI model groups were calculated using the Limma (linear models for microarray data) package. Gene sets with an expression of Log_2_ FC (Fold Change) > 1.5 and FDR (False Discovery Rate) < 0.05 were filtered for and collected. The collected GEO data were marked as *T_GEO_* (Barrett et al., 2007; Cappuzzello et al., 2009; Song et al., 2013; Steudemann et al., 2013; Gray et al., 2014; Liu et al., 2014, 2015, 2017; Zhang et al., 2014a,b; Peterson et al., 2015; Wang et al., 2015; Andersen et al., 2017; Gupta et al., 2017; Wu et al., 2017; Fang et al., 2018; Gunawan et al., 2018; Murugesan and Premkumar, 2018; Sabrkhany et al., 2018).

For the final step, the NCBI gene database^8^ was used to remove duplicates in the collected dataset and to normalize the official symbol in the *Mus Musculus* species. The union of targets collected from the open source database (*T_DB_*) and reference mining (*T_Ref_*) was then intersected with the DEG data (*T_GEO_*) mined from the GEO database (Figure 1B):

$$\begin{array}{c} T_{AMI}=(T_{DB}\cup T_{Ref})\cap T_{GEO} \end{array}$$

In which, *T_AMI_* refers to the target dataset of AMI.

##### Pharmacological effect correlated targets

**FIGURE 2:**
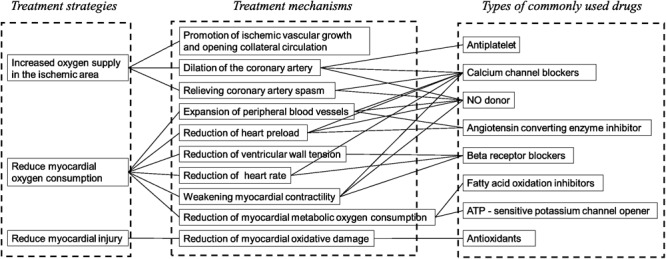
The clinical strategies and related mechanisms for the treatment of AMI. From eight clinically common drugs used in the treatment of AMI, the major therapeutic strategies of AMI can be classified into three categories: increase of the oxygen supply in the ischemic area, reduction of myocardial metabolism and protection of myocyte. The effect for the evaluation of *Schisandra* in the treatment of AMI can be selected: dilation of the artery, decrease of the heart afterload, decrease of the ventricular wall tension, decrease of the heart rate, weakening of the myocardial contractility, decrease of the myocardial metabolic oxygen consumption.

For the construction and analysis of a “gene ontology–effect” network, the targets related to pharmacological effects were screened according to the authoritative classification and references (Figure 2). The treatment mechanisms that were suitable for the network analysis and experimental evaluation can be summarized as anticoagulation, vasodilation, glucose metabolism, lipid metabolism, reduction of heart preload, ventricular wall tension, heart rate and myocardial contractility (Holmes et al., 2011; Husted and Ohman, 2015). Target data of pharmacological effects were screened through literature mining of PubMed references. The effect related target data were enriched by the GO terms for the further construction of a network model.

#### Construction of Three-Mode Network

##### Construction of GOI network

Based on screened AMI targets, the GO were enriched through the DAVID database, and further integrated with each other to construct the GOI network (Figure 5A) using the EnrichmentMap^9^ plugin in Cytoscape version 2.8. Each node represented a gene set corresponding to a GO term, and the edges and its weight were displayed and calculated based on the number of overlapping genes between two GO terms. The overlap similarity coefficient between significant GO terms was calculated and the cutoff was set to 0.5 (Merico et al., 2010; Isserlin et al., 2014; Cui et al., 2015).

##### Construction of “component-gene ontology” network

To construct the “component–gene ontology” two-mode network, which was based on screened component related targets, the DAVID database^10^ was used to perform GO enrichment annotation based on screened component related targets. The relationship between components and GO terms (including biological process, metabolic function and cell component) were then constructed into a “component-gene ontology” network (Figure 5B). There are two types of nodes in this network, *Schisandra* components and GO terms. Also, there are two types of edges in this network, including GO interaction and a component-GO term. In order to cover the scale of genes contained in the GO terms and the significance of these genes, weight scores between the *Schisandra* component and GO terms were calculated based on the percentage of proteins in each GO term and the significance test *p-*value. The formula was as follows:

$$\begin{array}{c} W_{Cm}=norm\{norm(r_{m})+norm[-\log(p_{m})]\} \end{array}$$

In which, *W_Cm_* refers to the weighted score of the *Schisandra* component related GO term m; *r_m_* refers to the ratio of protein included in the GO term; *p*_m_ refers to the significance test *p*-value of the GO term; *norm* refers to the min-max normalization method which was used to standardize *x* within the dataset (Isler and Kuntalp, 2010; Lu et al., 2012; Benaliouche and Touahria, 2014; Dinc et al., 2014; Cao et al., 2016; Zhang et al., 2016). The formulas were as follows:

$$\begin{array}{c} norm=\frac{x-min}{max-min} \end{array}$$

The pathways of the *Schisandra* component that were related to the target dataset were enriched through the KEGG and Biocarta database (Sun et al., 2011; Shangguan et al., 2015). Pathway scores were calculated using a significance *p-*value and the content ratio of the related component (Atobe et al., 2015; Dunkelberger et al., 2017; Babu et al., 2018; Desfontaine et al., 2018). The formulas were as follows:

$$\begin{array}{c} Score_{pathway}=\sum_{i=1}^{k}\{norm(r)+norm[-\log(p)]\}\times R_{i} \end{array}$$

In which *R_i_* refers to the content ratio of component *i* compared to all the identified *k* components. The potential pathway of *Schisandra* in the treatment of AMI were sequenced and filtered according to the accumulate score.

##### Construction of “gene ontology–effect” network

Pharmacological effect related targets were also enriched into GO terms, and then integrated into a “gene ontology-effect” two-mode network (Figure 5C). This network contained two types of nodes including GO terms and effects, and two types of edges; GO-GO interaction and GO-effect interaction. Weighted scores between the effect and GO terms were calculated based on the percentage of proteins in each GO term and the *p*-value. The formula was as follows:

$$\begin{array}{c} W_{En}=norm\{norm(r_{n})+norm[-\log(p_{n})]\} \end{array}$$

In which, *W*_En_ refers to the weighted score of the effect related GO term n; *r_n_* refers to the ratio of protein included in the GO term; *p*_n_ refers to the significance test *p*-value of the GO term; norm refers to the range method which was used for normalization.

##### Integration of “component–gene ontology–effect” three-mode network

In order to reduce the prediction bias and highlight the functional relation, the *Schisandra* components and pharmacological effects were connected through an AMI related GOI network, which represented functional interactions in the condition of myocardial infarction. The “component-gene ontology-effect” three-mode network (Figure 5D) was constructed through the integration of a “component-gene ontology” network and “gene ontology-effect” network. This three-mode network can be utilized to simulate and predict the action level and pharmaceutical mechanisms of the *Schisandra* component in the treatment of AMI related effects.

#### Analysis of Three-Mode Network

It would be feasible to evaluate the intensity of pharmacological effects in the disease condition, using a maximum flow algorithm based on the constructed three-mode network. A maximum flow algorithm was therefore carried out using Pajek software to calculate the score of each component related to effects based on the “component-gene ontology-effect” three-mode network (Looijestijn et al., 2015). The result scores were then adjusted using an entropy algorithm (Prado-Prado et al., 2011; Teschendorff et al., 2014; Hayasaka et al., 2015; Zhao et al., 2015), and the information entropy of effect *j* was defined as *H_j_*:

$$\begin{array}{c} H_{j}=-k\sum_{i=1}^{m}z_{ij}\cdot \ln z_{ij},\;j=1,2,3,...,n\\ k=\frac{1}{\ln m} \end{array}$$

In which, *Z_ij_* refers to the score calculated by the maximum flow algorithm, *i* infers the component *i* in all m components, *H_j_* refers to the information entropy of effect *j* in *n* effects.

The entropy weight *ω_j_* of the index *j* is defined as:

$$\begin{array}{c} \omega_{j}=\frac{1-H_{j}}{n-\sum_{j=1}^{n}H_{j}}\;j=1,2,3,...,n \end{array}$$

The predicted weight of the *Schisandra* components correlated to nine effects were calculated as:

$$\begin{array}{c} W_{j}=z_{ij}\cdot \omega_{ij} \end{array}$$

The score was then adjusted with the content ratio of each component:Score = W_ij_×R_i_, and the final score of the *Schisandra* component or the effect, based on the three–mode network, was calculated with the sum of the responsible adjusted score in the matrix:

$$\begin{array}{c} Score_{effect}=\sum_{j=1}^{n}w_{j}\\ Score_{component}=\sum_{i=1}^{m}w_{i} \end{array}$$

*W_i_* refers to the score of component *i* in all *m* components, *W_j_* refers to the score of effect *j* in all *n* effects.

### Experimental Validation

#### Surgery of LAD Ligation Model

The model of AMI was established by ligating the left anterior descending artery (LAD) of ICR mice (Gao et al., 2010; Borst et al., 2011) that weighed approximately 25 g (Laboratory Animal Unit of Qinglongshan, China), and hearts of the Sham group were operated without ligation. All mice that survived the surgery were randomly divided into the *Schisandra*, and the positive control group. According to the previous research based on “Yiqifumai” (Tasly, China) (Li et al., 2015) in the treatment of coronary heart disease, the experiment doses of *Schisandra* were set to a low dose of 0.056 g/kg, a medium dose of 0.23 g/kg and a high dose of 0.90 g/kg. In order to evaluate the unknown therapeutic effect of *Schisandra* on AMI in an unbiased way, five commonly used clinical drugs including Amlodipine (1601001, Yangzijiang, China) 1.52 mg/kg, Metoprolol (1703001, AstraZeneca) 15.17 mg/kg, Captopril (15022511, Changzhou, China) 7.58 mg/kg, Trimetazidine (2008516, Servier) 9.10 mg/kg were provided as the positive controls. Since the pharmacological mechanisms of these five positive drugs are different, they were only appended as the positive control when the pharmacological indicators were directly related to the corresponding mechanism of the positive drugs. All corresponding drugs were administered 24 h after the model was established, and the measurements were carried on 72 h after surgery (Figure 1C).

#### Echocardiography and Blood Pressure Analysis

Heart echocardiograms and blood pressure were measured 24 h after the mice received the corresponding drugs. Mice were anesthetized with isoflurane, placed on prewarmed trays, and maintained at normothermic levels during the examination. Parasternal long axis view-dependent M-mode and 2-D echocardiographic studies for determination of cardiac left ventricular hypertrophy were performed using a 55 MHz linear array transducer system (Vevo 770). Blood pressure was measured and assessed as the mean value of thirty consecutive measurements using a tail-cuff sphygmomanometer under unstressed conditions (BP-2000; Visitech, United States) (Sakata et al., 2017).

#### Histological Analyses

The heart tissues were merged into 4% formalin for 3 days and PBS with 30% sucrose for 1 day, then the tissues were embedded by an OCT compound and sliced into 10 μm using a freezing microtome. The slides with heart tissues were stained sequentially with eosin Y and hematoxylin Gill No. 3 for HE staining (201611, Jiancheng Bioengineering Institute, China). The pathological score of each sample was then evaluated based on the degree of necrosis and inflammation in the heart tissue.

#### Detection of Serum Biomarkers

Mice were sacrificed 48 h after administration. The blood serum of mice in each group were centrifuged at 3000 rpm for 10 min and then stored at room temperature within 20 min. The contents of lactate dehydrogenase (LDH) and free fatty acid (FFA) in the serum were detected through a test kit (201703, Jiancheng Bioengineering Institute, China). Serum noradrenaline (NE), angiotonin II (Ang II), NT and brain natriuretic peptide (NT-BNP), were detected using an enzyme-linked immunosorbent assay (ELISA) kit (201711, Jiancheng Bioengineering Institute, China). Each experiment was performed three times.

#### Immunocytochemistry

For the immunostaining experiments, slides with heart tissue were rinsed three times through PBS. Tissue was blocked for 1 h at 4°C in blocking buffer (PBS, 5% BSA, and 0.2% Triton). The slides were then incubated for 12 h at 4°C in *NFAT* or *NCOR2(SMRT)* antibodies against mouse (Proteintech Technology, China), which was diluted with TBST and mixed with 3% BSA. Slides were rinsed three times in PBS, followed by incubation in Alexa Fluor 488 secondary antibody conjugate (Beyotime, China). After incubation with DAPI for 5 min and the final rinse in PBS, slides were viewed under a fluorescence microscope.

#### Blood Flux and Clotting Time Measurements

##### The construction of coagulopathy model

Male ICR mice were randomly divided into six groups, including the Control, Model, *Schisandra* (Low: 0.056 g/kg, Middle: 0.23 g/kg, High: 0.90 g/kg) and Aspirin (BJ26934, Bayer, Italy) 15.17 mg/kg. Each mouse in the model and treatment group was injected with 0.08 ml/kg Adrenaline (ADR, Sigma–Aldrich). The mice were placed in water (4∼6°C) for 4 min after being injected with Adr, and were then injected again with 0.08 ml/kg Adr for a second time. The corresponding drug was administrated after injection for the second time. All these procedures were repeated for 3 days.

##### The blood flux measurement

The blood flux was measured 30 min after the last administration. Mice were anesthetized with chloral hydrate and prostrated on the trays. The Moor Laser Doppler Imaging device (Moor LDI, Moor Instruments Ltd, Axminster, Devon, United Kingdom) was utilized to measure blood flow (Blood Flux) in the micro vessels of the ears. The device was calibrated following the manufacturer’s guidelines. A recording of approximately 2 min for each sample was made, using both digital and graphic modes. For each measurement, a noise-free recording of approximately 5 s was used to calculate the average flux. The output data was then analyzed using the manufacturer’s software (Moorsoft v2.0) (Opazo Saez et al., 2005; Clough et al., 2009; Peeters et al., 2012).

##### Clotting time measurement

For each mouse, 100 μL blood was adopted through capillary from retrobulbar venous plexus, and the clotting time was evaluated at the time an obvious agglutination was identified (Li X. et al., 2013; Chapin and Hajjar, 2015; Chen et al., 2015).

#### Vasoconstriction Measurements

##### Animals and tissue preparation

Male Sprague-Dawley rats weighing between 250 and 300 g (Laboratory Animal Unit of Qinglongshan, China) were anesthetized 50 mg/kg, i.p. with Chloral hydrate. Rats were sacrificed through stunning and cervical dislocation, and the thoracic aorta was surgically removed. The aorta was cleaned of any adhering fat and connective tissues and cut into 3 mm wide rings (Li Y. et al., 2013; Baharara et al., 2014; Dai et al., 2017).

##### Isometric tension measurement

The aortic rings were immediately immersed in 37°C Krebs solution (NaCl 118 mM, KCl 4.7 mM, CaCl_2_ 2.5 mM, MgSO_4_ 1.2 mM, KH_2_PO_4_ 1.2 mM, NaHCO_3_ 25 mM, and Glucose 11.1 mM) after removal and aerated with 5% CO_2_/95% O_2_. Then the aortic rings were connected to a force transducer (Technology Co, Ltd., Chengdu, China) and stretched progressively to 2.0 g. All changes in tension were expressed as a percentage decrease in the contraction activated through 1 μM Adr after being mixed with Schisandra extracts with a dose between 9.0 × 10^-20^g/mL to 9.0 × 10^-04^ g/mL.

#### Statistical Analysis

Statistical differences were assessed by one way-ANOVA. *p* < 0.05 was considered statistically significant. Data were expressed as the mean ± SD.

## Results

### Pharmacological Effect Analysis of *Schisandra* Based on a “Component–Gene Ontology–Effect” Three-Mode Network

#### Data Mining of Targets Related to *Schisandra* Components

**FIGURE 3:**
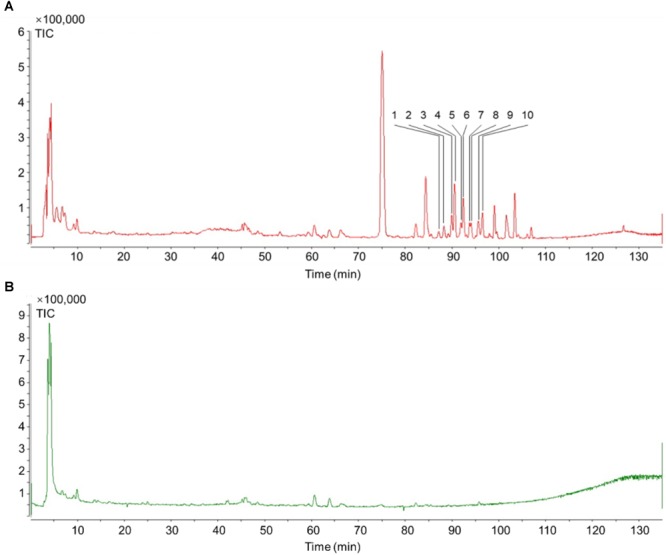
TIC of *Schisandra* extracts by HPLC-Q-TOF-MS-MS. **(A)** Positive ion mode MS spectra of *SchisandraSchisandra chinensis;* **(B)** Negative ion mode MS spectra of *SchisandraSchisandra chinensis.*

**FIGURE 4:**
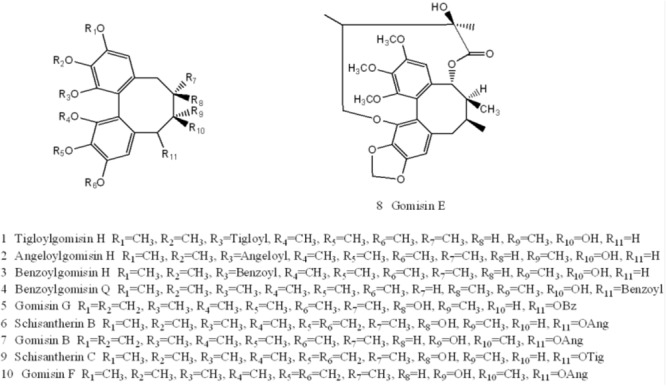
Structures of ten components identified in *Schisandra* extract: Tigloylgomisin H (1), Angeloylgomisin H (2), Benzoylgomisin H (3), Benzoylgomisin Q (4), Gomisin G (5), Schizantherin B (6), Gomisin B (7), Gomisin E (8), Schizantherin C (9), Gomisin F (10).

Ten components, including Tigloylgomisin H, Angeloylgomisin H, Benzoylgomisin H, Benzoylgomisin Q, Gomisin G, Schisantherin B, Gomisin B, Gomisin E, Schizantherin C, and Gomisin F, were identified from the water extracts of *Schisandra* (Figure 3, 4 and Supplementary Table S1) (Li et al., 2006; Wang et al., 2011). Pathway enrichment of target data based on identified components showed that “*NFAT* and Hypertrophy of the heart” and “Map Kinase Inactivation of *SMRT* Corepressor” were the top two correlated pathways of *Schisandra* in the treatment of AMI (Table 1 and Supplementary Tables S2, S3).

**Table 1:** The top 10 pathways related to *Schisandra* in the treatment of AMI.

Pathways	Score
*NFAT* in the Hypertrophic Cardiomyopathy	0.1558
Map Kinase Inactivation of *SMRT* (*NCOR2*) Corepressor	0.1369
Nuclear Receptors in Lipid Metabolism and Toxicity	0.1293
Phosphoinositides and their downstream targets.	0.0872
Aspirin Blocks Signaling Pathway Involved in Platelet Activation	0.0837
Bioactive Peptide Induced Signaling Pathway	0.0796
IL 2 signaling pathway	0.0794
BCR Signaling Pathway	0.0777
Inhibition of Cellular Proliferation by Gleevec	0.0624
Links between Pyk2 and Map Kinases	0.0623

#### Construction and Analysis of “Component–Gene Ontology-Effect” Three-Mode Network

Through mining of AMI related genes from three different data sources, the GEO data displayed the largest scale compared to the other two data sources (Figure 1B). After combining the target data from the database and intersection with GEO data, 3,288 DEG s from the heart tissue of *Mus Musculus* were confirmed (Figure 1B and Supplementary Tables S4, S5).

**FIGURE 5:**
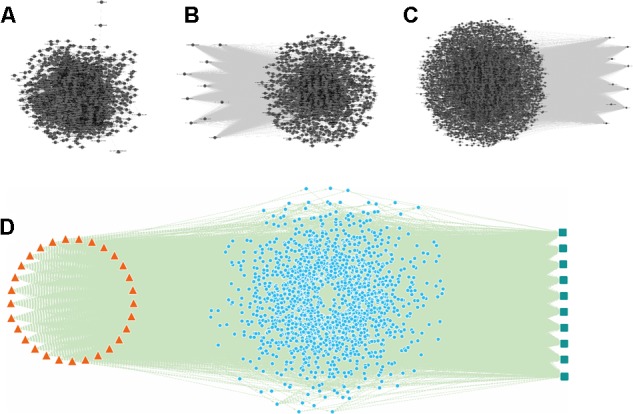
The “component-gene ontology-effect” three-mode network of *Schisandra*. Firstly, the AMI target related gene ontology interaction (GOI) network **(B)** was constructed by mining and enrichment of AMI related target genes. Secondly, the target proteins screened through identified *Schisandra* components were enriched into GO terms. The interactions between GO and the component were constructed and visualized through the “component-gene ontology” two-mode network **(A)**. Then, the “gene ontology-effect” two-mode network **(C)** for the treatment of AM pharmacological effects was constructed from the target data collected from reference mining. Finally, the three sub-networks were integrated, and the same GO nodes were combined to obtain a “component-gene ontology-effect” three-mode network **(D)**. The triangle in orange represents the components identified from *Schisandra*, the blue circle represents the common GO which are both relevant to the components and effects (green square).

**FIGURE 6:**
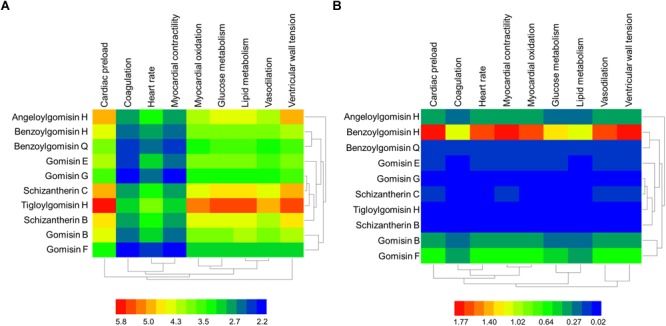
The predicted score heatmap of the *Schisandra* components related to the effects based on three-mode network. **(A)** The score matrix calculated from the three-mode network; **(B)** The score matrix adjusted with the content ratio of *Schisandra* components.

The GO terms enriched from collected AMI targets were connected with each other in order to integrate them into the GOI network (Tarnavski et al., 2004; Hunt et al., 2012; Tsukamoto et al., 2013). The “component-gene ontology-effect” three-mode network (Figure 5D) was then constructed by integrating the “component-gene ontology” network (Figure 5A), GOI network (Figure 5B) and the “gene ontology-effect” network (Figure 5C and Supplementary Tables S6, S7). The predicted score matrix of the *Schisandra* component related to the AMI effect was visualized by a heatmap (Figure 6). And the result indicated that cardiac preload and myocardial contractility were the top two pharmacological effects of the *Schisandra* components (Table 2), while Benzoylgomisin H was the top active component among all the identified components in the treatment of AMI (Table 3).

**Table 2:** Predicted score of effects based on the *Schisandra* components in the treatment of AMI.

Effect	Score
Cardiac preload	4.430
Ventricular wall tension	4.231
Myocardial contractility	4.133
Vasodilation	4.049
Heart rate	3.973
Myocardial oxidation	3.934
Glucose metabolism	3.186
Lipid metabolism	2.712
Coagulation	2.690

**Table 3:** Predicted score of the *Schisandra* components in the treatment of AMI.

Component	Network Score	Adj. Area	Score
Benzoylgomisin H	34.038	0.420	14.281
Gomisin F	27.593	0.202	5.578
Gomisin B	35.144	0.107	3.761
Angeloylgomisin H	37.867	0.098	3.705
Benzoylgomisin Q	31.126	0.063	1.965
Gomisin E	33.537	0.048	1.600
Schizantherin C	39.292	0.030	1.169
Tigloylgomisin H	44.512	0.017	0.760
Gomisin G	30.333	0.010	0.314
Schizantherin B	38.003	0.005	0.208

*Network Score: the score of components calculated through network result matrix; Adj. Area: the adjusted peak area of molecular ion related to each component; Score: the score adjusted through network analysis result and component peak area.*

### Experimental Validation of Network Analysis Results

#### Pharmacological Effect of *Schisandra* in the Treatment of AMI

**FIGURE 7:**
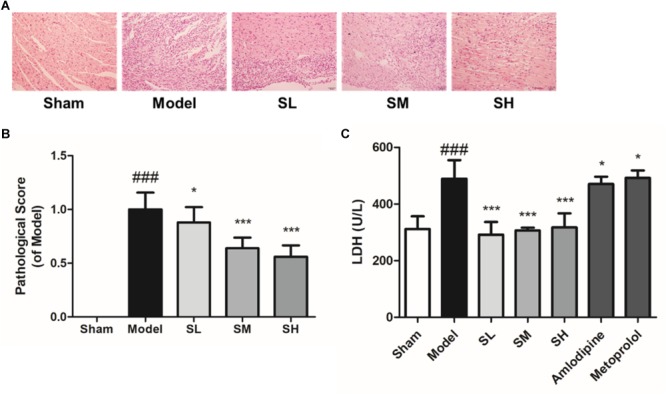
The histopathological staining and serum LDH of AMI mice treated with *Schisandra*. Sham group: subjected to the same surgical treatment without ligation, SL group: *Schisandra* extract was given to mice at the dose of 0.056 g/kg, SM group: *Schisandra* extract was given to mice at the dose of 0.23 g/kg, SH group: *Schisandra* extract was given to mice at the dose of 0.90 g/kg. **(A)** Mice heart sections were stained with H&E after given corresponding drug for two days (200×); **(B)** Pathological scores of H&E staining; **(C)** Serum LDH was tested after 2 days treatment of *Schisandra*. All data are shown as the mean ± SD. ^###^*P* < 0.001 vs. Sham group; ^∗^*P* < 0.05, ^∗∗∗^*P* < 0.001 vs. Model group.

The pathological analysis results showed that granulocytes were infiltrated into the heart tissue of the left ventricle and there was significant necrosis after LAD ligation surgery. *Schisandra* can alleviate the necrosis of the left ventricle in a dose-dependent way (Figure 7A,B). The serum level of LDH also indicated that *Schisandra* can significantly reduce the necrosis of myocytes after AMI (Figure 7C).

**FIGURE 8:**
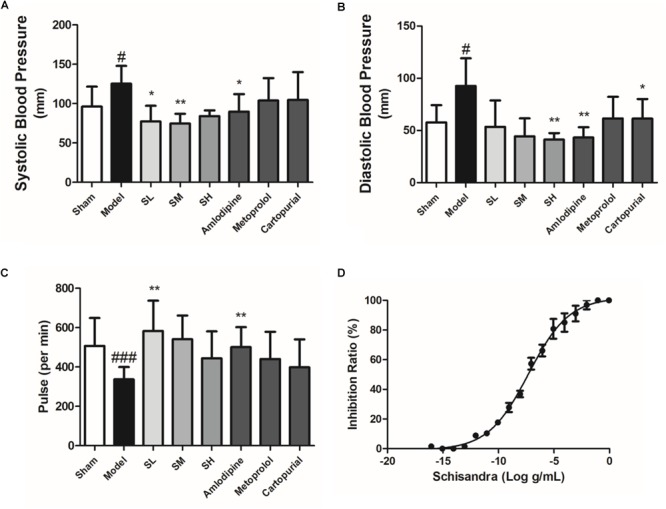
The blood pressure, heart rate and vasodilation ratio of *Schisandra*. Systolic blood pressure **(A)**, diastolic pressure **(B)**, and pulse **(C)** was tested 30 min after the last corresponding drug was given. **(D)** The vasodilation effect of *Schisandra*. The rat main artery was cut into 3 mm artery rings and were activated through 1 μM Adr. All data are shown as the mean ± SD. #*P* < 0.05, ###*P* < 0.001 vs. Sham group; ^∗^*P* < 0.05, ^∗∗^*P* < 0.01 vs. Model group.

*Schisandra* extract also showed a significant depressor effect both on the systolic blood pressure and diastolic blood pressure (Figure 8A,B). The pulse was elevated after the treatment of *Schisandra* in a negative feedback (Figure 8C). Studies of an adrenaline-induced artery ring model *in vitro* showed that *Schisandra* extract has a vasodilation effect at an EC_50_ dose of 5.144 × 10^-11^ g/mL (Figure 8D), which could partly explain the depressor effect of blood pressure.

**FIGURE 9:**
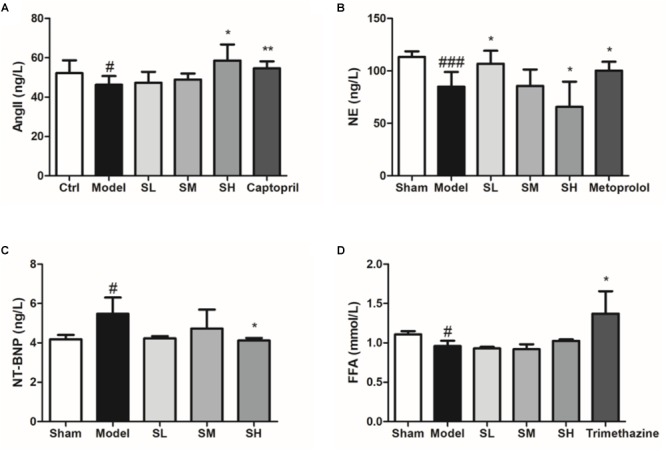
Serum Ang II **(A)**, NE **(B)**, NT-BNP **(C)**, and FFA **(D)** of *Schisandra* treated AMI mice. All data are shown as the mean ± SD. #*P* < 0.05, ###*P* < 0.001 vs. Sham group; ^∗^*P* < 0.05, ^∗∗^*P* < 0.05 vs. Model group.

The content of serum Ang II indicated that *Schisandra* could significantly elevate the content of serum Ang II (Figure 9A), which may be responsible for the vasodilation effect. On the other hand, serum NE was significantly reduced after LAD ligation surgery, whereas *Schisandra* could significantly reduce serum NE in a dose-dependent way, indicating that *Schisandra* could inhibit the sympathetic nervous system (Figure 9B). Additionally, the serum NT-BNP was significantly reduced after treatment with *Schisandra* (Figure 9C), which suggests that left ventricular wall tension and blood pressure can be significantly reduced (Demirtas et al., 2018; Pala et al., 2018; Shen et al., 2018; Xiong et al., 2018). These results indicate that *Schisandra* could significantly inhibit the activation of the sympathetic nervous system so that the contractility of the left ventricle could also be reduced. Additionally, the serum FFA result showed that *Schisandra* failed to condition the lipid metabolic function of myocytes during the 3 days of treatment (Figure 9D), and the immunofluorescence result of CPT1 also showed the same trend (Supplementary Figure S1). This result indicates that the conditioning of metabolism is not the major pharmacological effect of *Schisandra*.

**FIGURE 10:**
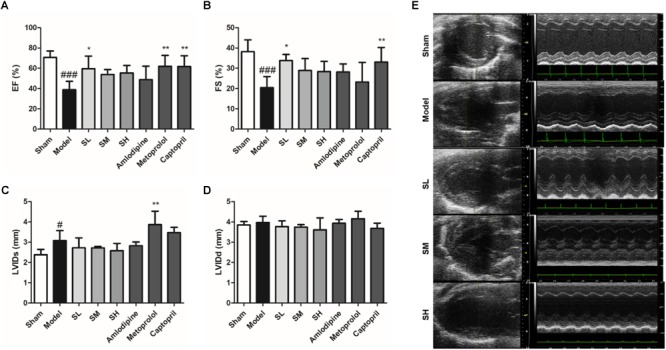
The EF **(A)**, FS **(B)**, LVIDs **(C)**, and LVIDd **(D)** result of *Schisandra* treated AMI mice. **(E)** The Ultrasonic Cardiogram of *Schisandra* treated mice was tested 30 min after the last corresponding drugs were given. All data are shown as the mean ± SD. #*P* < 0.05, ###*P* < 0.001 vs. Sham group; ^∗^*P* < 0.05, ^∗∗^*P* < 0.01 vs. Model group.

The ultrasonic cardiogram (US) result showed that *Schisandra* could significantly increase the ejection factor (EF) and fractional shortening (FS) of the AMI heart at a low dose, whereas the heart output and diameter in the left ventricle (LVIDd, LVIDs) was not affected by *Schisandra* after AMI (Figure 10A–E). It could be inferred from the result of BP and US that *Schisandra* can release heart burden by reducing myocardial contractility and increasing heart output through vasodilation.

**FIGURE 11:**
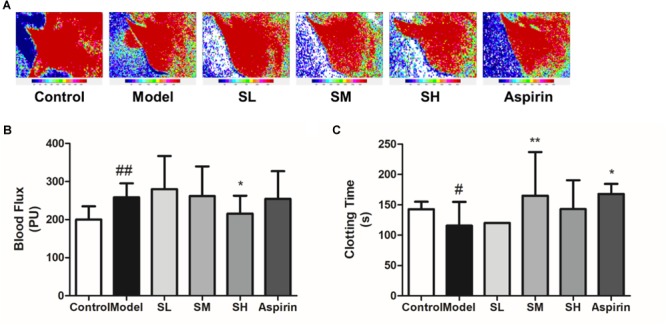
The blood flux **(B)** and clotting time **(C)** of AMI mice after treatment with *Schisandra*. **(A)** The mice were injected with Adr and then stimulated through 4°C water. After repeating these procedures twice, the FLPI was used to test blood flow in the ear vein. Then one drop of blood was taken for the test of clotting time by counting the seconds when the blood started to coagulate. All data are shown as the mean ± SD. #*P* < 0.05, ##*P* < 0.01 vs. Sham group; ^∗^*P* < 0.05, ^∗∗^*P* < 0.01 vs. Model group.

The blood flux results also indicate that *Schisandra* may induce the inhibition of sympathetic nerves and the subsequent downregulation of blood pressure (Figure 11A,B). On the other hand, *Schisandra* can significantly prolong the clotting time on the coagulopathy model (Figure 11C), which means it will lead to an increase of blood flow.

In conclusion, *Schisandra* extract showed significant cardiac inhibition and vasodilation effects, which further induces a decrease of heart burden and further preserves the heart contractility. These major pharmacological effects showed the same trend as the predicted results of the three-mode network.

#### NFAT and NCOR2 in Heart Tissue After Treatment With *Schisandra*

**FIGURE 12:**
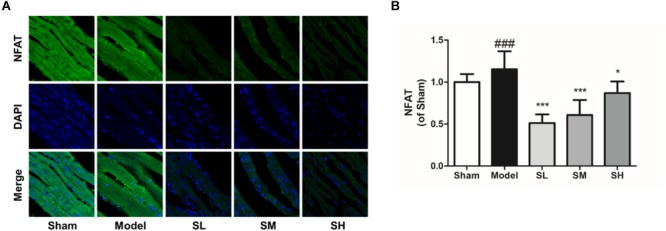
The immunofluorescence of *NFAT* in the heart tissue after the treatment of *Schisandra*. **(A)** The immunofluorescence staining of *NFAT* in heart tissue (400×); **(B)** The quantitation of *NFAT* from immunofluorescence staining results. All data are shown as the mean ± SD, ###*P* < 0.001 vs. Sham group; ^∗^*P* < 0.05, ^∗∗∗^*P* < 0.001 vs. Model group.

**FIGURE 13:**
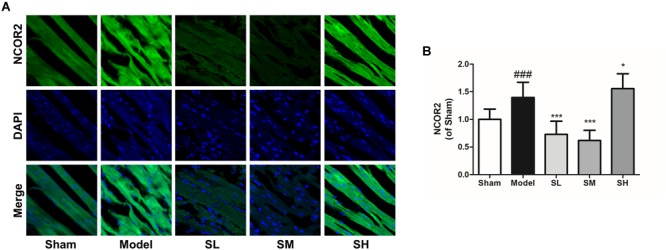
The immunofluorescence of *NCOR2* in the heart tissue after the treatment of *Schisandra*. **(A)** The immunofluorescence staining of *NCOR2* in heart tissue (400×); **(B)** the quantitation of *NCOR2* from immunofluorescence staining results. All data are shown as the mean ± SD, ###*P* < 0.001 vs. Sham group; ^∗^*P* < 0.05, ^∗∗∗^*P* < 0.001 vs. Model group.

Immunofluorescence staining result showed that the *NFAT* expression increased after the onset of AMI, and *Schisandra* could significantly reduce *NFAT* expression in myocytes (Figure 12A,B), whereas the inhibition effect was attenuated at a high dose. The expression of *NCOR2* in the heart tissues indicated that the pathologically reduced heart output led to an upregulation of *NCOR2* expression in myocytes after the onset of AMI (Kawamiya et al., 2010), and *Schisandra* could efficiently inhibit the over-expression of *NCOR2* in myocytes (Figure 13A,B).

## Discussion

Network pharmacology is an effective way to predict the complex mechanisms of TCM and has widely been used in the prediction of pharmacological mechanisms in order to guide pharmacological research. Thus, the development of a new network analysis methodology with higher accuracy and based on an open source high-throughput dataset, will support the pharmacological research of TCM in a much more convenient and economical way. However, it remained difficult to predict and evaluate the relationship between the component and pharmacological effect of the multi-drug system in a systematical way due to the complex mechanisms between proteins. Thus, it would be more valuable to clarify the pharmacological function of the drug rather than just filtering one target protein to guide further pharmacological research. Generally speaking, it would be more reasonable to highlight the pharmacological functional characteristics of the network model rather than a single target prediction, and the GO enrichment could solve this problem.

In this research, the target data was collected from public databases, reference literature mining and GEO array data to ensure that the collected data included the latest research results and to ensure that the expression difference significance in the heart tissue of *Mus Musculus* had been experimentally verified. Instead of component related target proteins only, the GO was used for the enrichment of drug-related targets in order to reduce the false positive prediction error of target prediction and to highlight the structural-functional characteristics of the drug components. The “component–gene ontology–effect” three-mode network, based on the GOI network, which represented the pharmacological functional signal pathway stream between the drug component and phenotype effect, was then integrated and the maximum flow algorithm was used to analyze the pharmacological functional message flow between the components and effects. By using this method, the phenotype effect of a complex system could be evaluated.

After the onset of AMI, the necrosis of myocytes in the left ventricle led to a decrease of contraction force. The decreased heart output then induced an increased amount of blood retention in the left ventricle, which further increased the heart burden and oxygen consumption. The hypoxia of myocytes leads to apoptosis and fibration in the long term (Herum et al., 2015; Lighthouse and Small, 2016). According to the results of the network analysis, a reduction of cardiac preload and myocardial contractility was the major effect of *Schisandra* in the treatment of AMI. The further experimental validation also proved that, compared to other effects, *Schisandra* could significantly reduce the blood pressure through the inhibition of the sympathetic nerves, which led to a decrease of heart preload and the myocyte ischemia and apoptosis could be reduced.

The immunofluorescence results confirmed that *Schisandra* components in the treatment of AMI were related to *NCOR2* and *NFAT*. Research has shown that *SMRT* (*NCOR2*) can cooperate with thyroid hormone receptor-α (TRα) and up-regulate the expression of *CaMK II*, which leads to an unregulated sensitivity with sympathetic nerve activation (Kawamiya et al., 2010; Barish et al., 2012; Xie et al., 2015). After the onset of AMI, *NCOR2* expression was decreased according to the references. However, experiment results in *Mus Musculus* showed that the increased expression of *NCOR2* after LAD ligation surgery may be induced by negative feedback of reduced heart output at the onset of ischemia. The *Schisandra* could significantly reduce the expression of *NCOR2*, which may induce the negative effect of heart burden. However, research has shown that the Ca^2+^-calcineurin-*NFAT* pathway was activated due to the activation of the β-receptor after the onset of AMI. The increased expression of *NFAT* induces the reconstruction of the left ventricle, leading to heart failure in the long term after AMI. *Schisandra* could inhibit the expression of *NFAT* (Lee et al., 2003; de Salvi Guimaraes et al., 2017), which may inhibit fibrosis in the long term, so the output function of the heart will be preserved. However, further research needs to be conducted for complete experimental validation of *NCOR2* and *NFAT* pathways related to *Schisandra*.

In general, the “component-gene ontology-effect” three-mode network could be a proper model to analyze effects, based on a multi-drug complex system, in a systematical way. This network analysis and experiment validation workflow may provide a reasonable method to research multi-drug systems including TCM.

## Data Availability

Publicly available datasets were analyzed in this study. This data can be found here: http://www.genecards.org.

## Author Contributions

SH carried out this research with the help of HZ and the guidance of JQ, YH, and BY.

## Conflict of Interest Statement

The authors declare that the research was conducted in the absence of any commercial or financial relationships that could be construed as a potential conflict of interest.

## Footnotes

1 lilab.ecust.edu.cn/PharmMapper/

2 http://www.genecards.org/

3 https://www.pharmgkb.org/

4 http://bidd.nus.edu.sg/group/ttd/

5 http://www.ncbi.nlm.nih.gov/pubmed/

6 http://www.ncbi.nlm.nih.gov/geo

7 http://www.bioconductor.org/

8 https://www.ncbi.nlm.nih.gov/gene/

9 http://www.baderlab.org/Software/EnrichmentMap

10 https://david-d.ncifcrf.gov

## Supplementary Material

FIGURE S1 The immunofluorescence of CPT1 in the heart tissue after the treatment of *Schisandra*. **(A)** The immunofluorescence staining of CPT1 in heart tissue (400 ×); **(B)** The quantitation of CPT1 from immunofluorescence staining results. All data are shown as the mean ± SD. ^###^*p* < 0.001 vs. Sham group.

TABLE S1 Components identified from *Schisandra* extract through HPLC-Q-TOF-MS-MS.

TABLE S2 Target data simulated through Pharmmapper based on identified *Schisandra* components.

TABLE S3 *Schisandra* related pathways enriched through simulated targets.

TABLE S4 AMI related targets collected through datamining.

TABLE S5 Gene ontology terms of AMI related targets.

TABLE S6 Targets related with pharmacological effects.

TABLE S7 Gene ontology terms of AMI pharmacological effects.
