# Supplementary material for: Analysis of Effect of Schisandra in the Treatment of Myocardial Infarction Based on Three-Mode Gene Ontology Network
Source: Front Pharmacol. 2019 Mar 20;10:232. doi: 10.3389/fphar.2019.00232 (PMC6435518; doi:10.3389/fphar.2019.00232)
Supplement: FIGURE S1 — The immunofluorescence of CPT1 in the heart tissue after the treatment of Schisandra. (A) The immunofluorescence staining of CPT1 in heart tissue (400 ×); (B) The quantitation of CPT1 from immunofluorescence staining results. All data are shown as the mean ± SD. ###p < 0.001 vs. Sham group. [file Image_1.pdf]

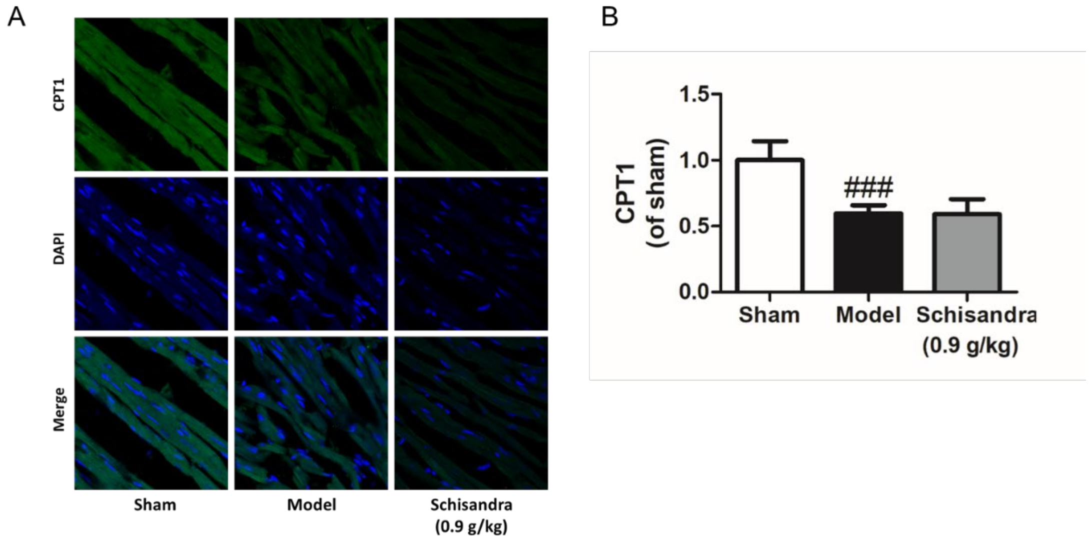

Supplementary Figure 1: The immunofluorescence of CPT1 in the heart tissue after the treatment of Schisandra. (A) The immunofluorescence staining of CPT1 in heart tissue(400 ×); (B) The quantitation of CPT1 from immunofluorescence staining results. All data are shown as the mean±S.D., ###, P<0.001 vs. Sham group.
