# Supplementary material for: Analysis of Effect of Schisandra in the Treatment of Myocardial Infarction Based on Three-Mode Gene Ontology Network
Source: Front Pharmacol. 2019 Mar 20;10:232. doi: 10.3389/fphar.2019.00232 (PMC6435518; doi:10.3389/fphar.2019.00232)
Supplement: TABLE S1 — Components identified from Schisandra extract through HPLC-Q-TOF-MS-MS. [file Table_1.DOCX]

Supplementary Table 1 Components identified from Schisandra extract

| Peak No. | Rt | Compound ID | Peak Area | [M+H]^+^ | Positive ions (m/z) |
| --- | --- | --- | --- | --- | --- |
| 1 | 85.5 | Tigloylgomisin H | 1477766 | 501 | 523.2309[M+Na]^+^ |
|  |  |  |  |  | 501.2486[M+H]^+^ |
|  |  |  |  |  | 483.2380[M+H-H_2_O]^+^ |
|  |  |  |  |  | 431.2208[M+H-C_5_H_10_]^+^ |
|  |  |  |  |  | 401.1953[M+H-C_5_H_8_O_2_]^+^ |
| 2 | 88.2 | Angeloylgomisin H | 8467057 | 501 | 523.2296[M+Na]^+^ |
|  |  |  |  |  | 501.2485[M+H]^+^ |
|  |  |  |  |  | 483.2374[M+H-H_2_O]^+^ |
|  |  |  |  |  | 431.2070[M+H-C_5_H_10_]^+^ |
|  |  |  |  |  | 401.1964[M+H-C_5_H_8_O_2_]^+^ |
| 3 | 90.6 | Benzoylgomisin H | 36312825 | 523 | 523.2298[M+H]^+^ |
|  |  |  |  |  | 505.2201[M+H-H_2_O]^+^ |
|  |  |  |  |  | 487.0850[M+H-2H_2_O]^+^ |
|  |  |  |  |  | 401.1960[M+H-C_7_H_6_O_2_]^+^ |
| 4 | 92.4 | Benzoylgomisin Q | 5463993 | 548 | 548.2861[M+H]^+^ |
|  |  |  |  |  | 431.2063[M+H-C_5_H_7_O]^+^ |
|  |  |  |  |  | 386.6472[M+H-C_6_H_7_O_3_]^+^ |
|  |  |  |  |  | 356.1541[M+H-C_6_H_10_O_3_-CO]^+^ |
| 5 | 92.6 | Gomisin G | 894753 | 537 | 537.2164[M+H]^+^ |
|  |  |  |  |  | 415.1786[M+H-C_7_H_6_O_2_]^+^ |
|  |  |  |  |  | 371.0599[M+H-C_7_H_6_O_2_-C_2_H_4_O]^+^ |
|  |  |  |  |  | 343.6104[M+H-C_7_H_6_O_2_-C_2_H_4_O-CO]^+^ |
| 6 | 93.1 | Schisantherin B | 472587 | 515 | 537.2097[M+Na]^+^ |
|  |  |  |  |  | 515.2276[M+H]^+^ |
|  |  |  |  |  | 415.1753[M+H-C_5_H_8_O_2_]^+^ |
|  |  |  |  |  | 371.0599[M+H-C_5_H_8_O_2_-C_2_H_4_O]^+^ |
| 7 | 94.0 | Gomisin B | 9261036 | 515 | 537.2083[M+Na]^+^ |
|  |  |  |  |  | 515.2274[M+H]^+^ |
|  |  |  |  |  | 415.2123[M+H-C_5_H_8_O_2_]^+^ |
| 8 | 95.5 | Gomisin E | 4129441 | 515 | 537.2082[M+Na]^+^ |
|  |  |  |  |  | 515.2281[M+H]^+^ |
|  |  |  |  |  | 385.0755[M+H-C_6_H_10_O_3_]^+^ |
| 9 | 95.7 | Schizantherin C | 2574143 | 515 | 515.2273[M+H]^+^ |
|  |  |  |  |  | 385.0752[M+H-C_6_H_10_O_3_]^+^ |
|  |  |  |  |  | 355.1729[M+H-C_7_H_12_O_4_]^+^ |
|  |  |  |  |  | 338.3420[M+H-C_7_H_12_O_5_]^+^ |
| 10 | 96.5 | Gomisin F | 17495514 | 515 | 537.2083[M+Na]^+^ |
|  |  |  |  |  | 515.2268[M+H]^+^ |
|  |  |  |  |  | 415.1752[M+H-C_5_H_8_O_2_]^+^ |
|  |  |  |  |  | 387.3726[M+H-C_5_H_8_O_2_-CO]^+^ |
